# The transcriptional network activated by Cln3 cyclin at the G1-to-S transition of the yeast cell cycle

**DOI:** 10.1186/gb-2010-11-6-r67

**Published:** 2010-06-23

**Authors:** Francisco Ferrezuelo, Neus Colomina, Bruce Futcher, Martí Aldea

**Affiliations:** 1 Departament de Ciències Mèdiques Bàsiques, Institut de Recerca Biomèdica de Lleida, Universitat de Lleida, Montserrat Roig 2, 25008 Lleida, Spain; 2 Department of Molecular Genetics and Microbiology, Stony Brook University, Stony Brook, NY 11794, USA

## Abstract

**Background:**

The G1-to-S transition of the cell cycle in the yeast *Saccharomyces cerevisiae *involves an extensive transcriptional program driven by transcription factors SBF (Swi4-Swi6) and MBF (Mbp1-Swi6). Activation of these factors ultimately depends on the G1 cyclin Cln3.

**Results:**

To determine the transcriptional targets of Cln3 and their dependence on SBF or MBF, we first have used DNA microarrays to interrogate gene expression upon Cln3 overexpression in synchronized cultures of strains lacking components of SBF and/or MBF. Secondly, we have integrated this expression dataset together with other heterogeneous data sources into a single probabilistic model based on Bayesian statistics. Our analysis has produced more than 200 transcription factor-target assignments, validated by ChIP assays and by functional enrichment. Our predictions show higher internal coherence and predictive power than previous classifications. Our results support a model whereby SBF and MBF may be differentially activated by Cln3.

**Conclusions:**

Integration of heterogeneous genome-wide datasets is key to building accurate transcriptional networks. By such integration, we provide here a reliable transcriptional network at the G1-to-S transition in the budding yeast cell cycle. Our results suggest that to improve the reliability of predictions we need to feed our models with more informative experimental data.

## Background

In the model yeast *Saccharomyces cerevisiae*, the commitment to a new round of cell division takes place towards the end of the G1 phase of the cell cycle, a process called START [1]. This entails the unfolding of a transcriptional program involving over 200 genes, including some important cell cycle regulators such as the G1 cyclins Cln1 and Cln2, S phase cyclins, a number of cell cycle transcription factors (TFs) as well as many other genes with functions related to DNA metabolism (replication, repair, and so on), budding, spindle pole body duplication, and cell wall synthesis [2,3]. Many of these genes are known or putative targets of two heterodimeric TFs called SBF and MBF. SBF contains the DNA-binding protein Swi4, while MBF contains the Swi4-related DNA-binding protein Mbp1, and both factors contain the regulatory protein Swi6, which binds directly to Swi4 or Mbp1, respectively (reviewed in [4]). There is considerable functional redundancy between these factors. For example, it has been reported that SBF may recognize, albeit with reduced affinity, MBF binding sites and vice versa [5-7]. Moreover, while *mbp1Δ *and *swi4Δ *strains are viable, the double mutant *mbp1Δ swi4Δ *is not [8].

Although MBF and SBF are poised at their target promoters during much of G1 phase [9-11], they cannot activate transcription; rather, they repress it. Their activation at START depends primarily on the cyclin/cyclin-dependent kinase (CDK) complex Cln3-Cdc28. This is achieved in part by phosphorylation, and consequent shuttling out of the nucleus, of a repressor called Whi5 [12,13], releasing SBF/MBF from its inhibition. Recently, a positive feedback mechanism involving Cln1 and Cln2 has been proposed to operate under physiological conditions in SBF/MBF activation [14].

There has been considerable interest and effort at elucidating TF-target interactions at a genome scale. Reliable TF-target assignments are essential to build accurate transcriptional networks and to uncover TF modules responsible for combinatorial transcriptional regulation. One important piece of information concerning TF-target assignments is provided by genome-wide location analyses of TFs [15-18]. However, TF binding does not necessarily imply regulation, neither is it informative as to whether the regulation is positive or negative. Furthermore, these studies are typically noisy, and given the modest overlap among some of these analyses, and the poor agreement with data from other sources, doubts about their reliability have also been raised [19,20]. Nonetheless, location analyses have been the starting point for numerous computational studies aimed at defining transcriptional networks by heterogeneous data integration (see, for instance, Lee *et al. *[21] and references therein). Following these lines, two recent works, one based on a Bayesian approach [22] and another using support vector machines [23], have provided predictions for TF-target interactions in the yeast global transcriptional network. Unfortunately, the agreement between these studies is at most quite modest.

We are particularly interested in the transcriptional program at START. In order to produce informative experimental data concerning this cell cycle stage, we have used DNA microarrays to generate new expression profiles under relevant conditions (synchronized cultures, deletion mutants) to study the transcriptional targets of the START regulator Cln3, and their dependence on the TFs Mbp1 and Swi4. We have integrated our new data with previously published datasets to provide reliable TF-target assignments. We propose a list of more than 150 targets. Importantly, we have experimentally validated our new predictions by performing chromatin immunoprecipitation (ChIP) to demonstrate TF binding to the promoters of some of our targets. Furthermore, our classification performs better than recent analyses [22,23] in a number of tests, and shows high internal consistency.

## Results

### New genome-wide expression dataset

**Figure 1:**
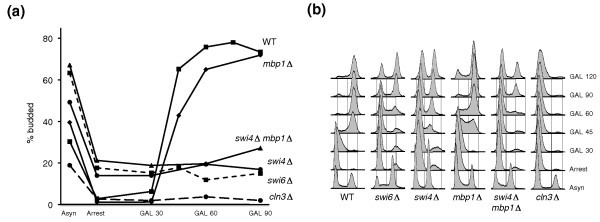
**Budding index and DNA content**. Relevant genotypes of strains are shown. Strains were also deleted for *BCK2*, and contained plasmid pRS313{P_*MET3*_*·CLN2*}. Except for control strain *cln3Δ*, all strains also had the endogenous *CLN3 *gene expressed under the *GAL1 *promoter. Asynchronous (Asyn) cultures of the indicated strains were grown in raffinose medium lacking methionine; they were thus kept alive by constitutive expression of *CLN2*. Cells were arrested in G1 (Arrest) by addition of methionine. After most cells were blocked in G1, galactose was added to induce *CLN3*. Samples were taken every 15 minutes for **(a) **budding and **(b) **DNA content evaluation (not all time points are shown). Only one experiment is shown. Somewhat less synchronous but otherwise similar profiles were obtained in a duplicate experiment (data not shown). WT, wild type.

In order to identify the targets of the cell cycle regulator Cln3, and their dependence on the TFs SBF and MBF, we have used DNA microarrays to interrogate genome-wide changes in gene expression upon induction of Cln3 in strains that lacked components of SBF, MBF or both, that is, *swi6Δ*, *swi4Δ*, *mbp1Δ*, and *swi4Δ mbp1Δ *mutants. Cln3 becomes essential in the absence of Bck2 [24-26]. Recently, we have also shown that overexpressed Bck2 is able to induce an extensive transcriptional program of mostly cell cycle-regulated (CCR) genes, many of which peak at the G1/S transition of the cell cycle [27]. Hence, to avoid confounding effects derived from Bck2 function, we placed the endogenous *CLN3 *gene under the control of the regulatable *GAL1 *promoter in strains deleted for *BCK2*. When grown under non-inducing conditions for the *GAL1 *promoter, P_*GAL1*_*·CLN3 bck2Δ *strains were kept alive by constitutive expression of *CLN2 *(pRS313{P_*MET3*_*·CLN2*} [26]). Also, to control for non-specific expression changes, we used a double deletion *cln3Δ bck2Δ *strain, again kept alive by P_*MET3*_*·CLN2*. To improve sensitivity and facilitate interpretation, before galactose induction we synchronized our cultures by repressing the expression of *CLN2 *with methionine. Cln2 depletion in a raffinose (non-inducing) medium produced a G1 arrest similar to that described for a *cln3Δ bck2Δ *double mutant [24-26], that is, accumulation of unbudded cells with 1N DNA content (Figure 1).

**Figure 2:**
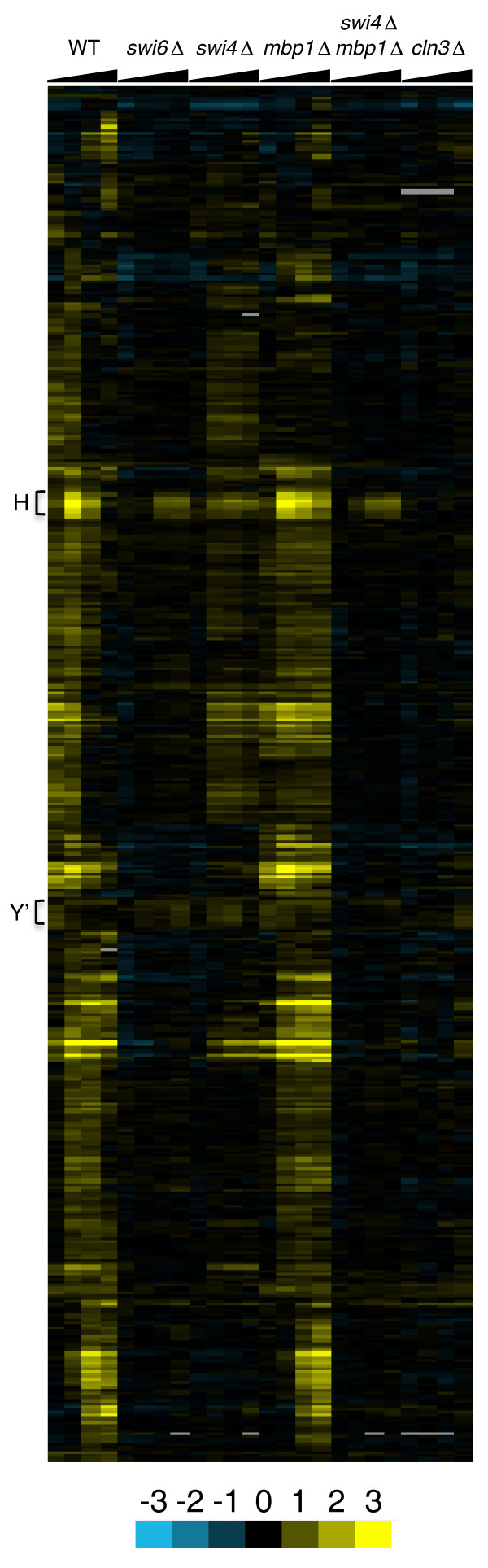
**Expression profiles of the 445 genes selected in this study**. Heat map depicting relative expression levels after galactose addition. Induction is yellow; repression is blue. Averaged log2 values from duplicate experiments are used (for individual values see Additional file 1). Scale is at the bottom. Only relevant genotypes of strains are indicated. For complete genotypes see Figure 1 or the text. Four time points (20 through 80) per strain are indicated by widening black bars at the top. Genes are hierarchically clustered (uncentered Pearson correlation, average linkage). On the left, H indicates the histone cluster; Y' indicates the cluster of Y' subtelomeric elements. WT, wild type.

Overexpressed *CLN3 *induced cell cycle entry in an *mbp1Δ *background and in an otherwise wild-type strain (that is, in a *bck2Δ *context), as assessed by DNA content and budding count. By contrast, Cln3 was unable to increase the budding index in *swi6Δ*, *swi4Δ *or *swi4Δ mbp1Δ *strains (Figure 1a). Interestingly, Cln3 was capable of promoting DNA replication in these backgrounds, even though it was unable to induce any noticeable changes in gene expression in the *swi6Δ *or *swi4Δ mbp1Δ *mutants (Figures 1b and 2). Most likely, this is due to overexpressed Cln3 being able to target the Clb/Cdc28 inhibitor Sic1 for degradation [28]. As expected, galactose addition *per se *was unable to induce cell cycle entry in the *cln3Δ bck2Δ *control strain (Figure 1).

Cultures were sampled every 20 minutes for the next 80 minutes after galactose addition, and changes in gene expression were measured using microarrays. In order to select genes specifically induced by Cln3 (or by cell cycle entry) as opposed to those induced by stress or by galactose, we used five slightly different selection criteria based on gene clustering (see Materials and methods). The number of genes selected by each criterion ranges from 225 to 327, totaling 445 genes, of which 144 (32%) were selected by all five approaches used, whereas 118 genes were selected by only one method. The expression patterns of all 445 candidate genes are shown in Figure 2 (see Additional file 1 for numerical values). We anticipated that because we used synchronized cultures, and because Cln3 is a key cell cycle regulator, most of these genes would be CCR. Indeed, more than 70% of the 445 genes selected are CCR. Importantly, this is true even when we did not use CCR gene enrichment as a selection criterion. Furthermore, most (68%) of these CCR genes peak at G1 or S phases of the cell cycle, as expected for Cln3 targets. Hence, it is likely that our microarray analysis has produced a meaningful set of putative Cln3 targets.

As we have reported before [27], virtually all genes are irresponsive to Cln3 in the absence of Swi6. Here, we also show that Cln3 requires either Mbp1 or Swi4 in order to promote transcription of its targets, as deduced from the lack of induction in the *swi4Δ mbp1Δ *strain. Hence, we demonstrate that Cln3 functions as a transcriptional regulator exclusively through MBF and SBF. The only genes that were somewhat induced in both the *swi6Δ *and *swi4Δ mbp1Δ *backgrounds were histones (Figure 2). Rather than indicating an MBF/SBF-independent Cln3-mediated induction, this is very likely due to ongoing DNA replication because histones are regulated at multiple levels and show a robust expression peak in S phase (reviewed in [29]). Another cluster of genes that also showed some induction in the absence of Swi6 contains helicases encoded by middle-repetitive Y' subtelomeric regions. Because there is extensive sequence similarity among these loci, it is unclear whether all reported features or just one or few were actually induced in our experiments. In any case, we also observed some induction of these genes in the control strain, albeit with different timing than in the other strains (Figure 2).

### Transcription factor-target assignments

**Figure 3:**
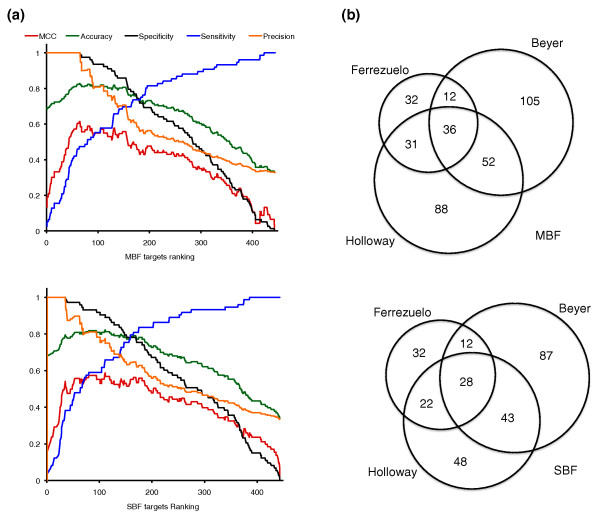
**Target classifications**. **(a) **Values of quality measures throughout our ranked list of candidates. Average values obtained with two benchmarks are represented. See text for details. **(b) **Venn diagrams comparing our classifications with those of Beyer *et al. *[22] and Holloway *et al. *[23].

To distinguish the targets of Cln3 from those genes that were just responding to cell cycle progression, and because we found that Cln3 functions exclusively through MBF or SBF, we determined the subset of genes within the 445 candidates that could be assigned to either MBF, SBF or both. To do this, we used a Bayesian approach that integrates different lines of evidence into a single probabilistic model [22,30]. In our analysis, we have evaluated nine different classifiers from three different lines of evidence - TF binding information, TF motifs, and expression data. For each classifier considered, each TF-target interaction was assigned a log-likelihood score based on control sets of positive and negative interactors. Final scores were computed by simply adding all the individual scores for the nine classifiers employed. These scores are provided in Additional file 2. To choose thresholds in our ranked list of putative targets, we evaluated our predictions with several statistical measures (Figure 3a). We selected cutoffs that at the same time produced high values of the Matthews correlation coefficient (MCC) [31] - regarded as a balanced measure of the quality (predictive power) of binary classifications, even when classes are of very different sizes - and also produced high values for accuracy (›80%), precision (›80%), and specificity (›90%); somewhat at the expense of sensitivity (approximately 60%). In other words, we preferred to leave out some true positives to avoid the inclusion of too many false positives. In any case, these quality values are likely underestimated (see Materials and methods).

By these criteria, we obtained 111 and 94 targets of MBF and SBF, respectively. Thirty-six of these were shared by both factors (Tables 1 and 2; Additional file 3). We first examined our predictions for targets for which strong evidence of regulation by MBF or SBF exists in the literature (reviewed in [32]) [19,33,34]. For this purpose, we avoided noisy datasets generated by genome-wide approaches. We found a total of 14 genes. Of the seven genes showing MBF regulation (*CDC21*, *POL1*, *CLB5*, *CLB6*, *RNR1*, *NRM1*, *DUN1*), our list of targets includes six. The only exception, *NRM1*, was ranked number 161. We classified *NRM1 *as an SBF target instead. Only one gene, *DUN1*, was in the positive control set. Similarly, of the seven reported targets of SBF (*HO*, *CLN1*, *CLN2*, *PCL1*, *SVS1*, *TOS4*, *YOX1*), we were able to detect all except *PCL1 *(position 165) as SBF-regulated genes. *HO *and *TOS4 *were in the positive control set. Hence, we conclude that our strategy correctly assigned most known targets of MBF or SBF. Among our predictions, 58% and 67% of the MBF and SBF targets, respectively, have also been reported in a number of previous analyses [35-38] other than Beyer's and Holloway's studies. This suggests that our approach has produced many true targets, as substantiated by independent classifications. On the other hand, we have predicted 27 MBF- and 21 SBF-regulated genes not found before [22,23,35-38]. Although this constitutes added value to our work, it raises questions about the number of false positives in our analysis, and it calls for further experimental validation of our results (see below).

**Table 1:** Summary of targets controlled by MBF

Ranking	Systematic name	Standard name	Cell cycle peak^a^	TF binding^b^	Motifs ACGCG^c^	Previous classifications^d^	Functional class^e^
1	YMR179W	*SPT21*	14	[15-18]	2 (1)	[23,35-38]	Others
2	YKL113C	*RAD27*	20	[15-18]	1 (1)	[23, 35-38]	DNA RRR
3	YLR103C	*CDC45*	18	[15-18]	2	[22, 23, 35-38]	DNA RRR
4	YNL102W	*POL1*	20	[15,18]	3 (1)	[22,23,36]	DNA RRR
5	YJL074C	*SMC3*	19	[17,18]	2	[22,23,35-38]	Cell cycle
6	YOR074C	*CDC21*	22	[15-18]	2	[22,23,35-38]	DNA RRR
7	YNL312W	*RFA2*	22	[15,17,18]	2	[22,23,36,37]	DNA RRR
8	YAR007C	*RFA1*	19	[17,18]	2 (1)	[22,23,36-38]	DNA RRR
9	YAR008W	*SEN34*	17	[17,18]	2 (2)	[22,23,36-38]	Others
10	YDL003W	*MCD1*	20	[15-18]	2 (2)	[23,35-38]	Cell cycle
41	YNL082W	*PMS1*	13		2		DNA RRR
56	YOR144C	*ELG1*	16		1		DNA RRR
66	YKL092C	*BUD2*	ND		1		BP
67	YDL157C		32		1		Unknown
68	YNL206C	*RTT106*	19		1		DNA RRR
69	YKL108W	*SLD2*	13		1		DNA RRR
70	YOR284W	*HUA2*	17		1		BP
71	YDL164C	*CDC9*	18		2	[36]	DNA RRR
72	YLR032W	*RAD5*	15		2		DNA RRR
77	YDL102W	*POL3*	17		1 (1)		DNA RRR
78	YNL263C	*YIF1*	24		1 (1)		Others
79	YPL208W	*RKM1*	23		1		Others
83	YKL042W	*SPC42*	21		1		SPB
84	YML133C		8	[15]	(1)		DNA RRR
85	YJL173C	*RFA3*	31		2 (1)	[36]	DNA RRR
86	YJL181W		13		3		Unknown
88	YKL089W	*MIF2*	ND			[36]	SPB
89	YML060W	*OGG1*	22		1		DNA RRR
90	YBR275C	*RIF1*	22	[18]			DNA CM
91	YOR368W	*RAD17*	ND		1		DNA RRR
95	YNL339C	*YRF1-6*	10	[15]	(1)		DNA RRR
96	YOL090W	*MSH2*	20		1	[36]	DNA RRR
99	YOR114W		24		1		Unknown
101	YHL013C	*OTU2*	ND	[17]		[35,37]	Unknown
103	YOR195W	*SLK19*	27		1		SPB
104	YGR140W	*CBF2*	34		1 (1)		Cell cycle
106	YNL309W	*STB1*	15		1 (1)		Cell cycle
107	YOL034W	*SMC5*	ND		1		DNA RRR
108	YER016W	*BIM1*	29		2		Cytoskeleton
109	YDR356W	*SPC110*	33		1		Cytoskeleton
110	YDL105W	*NSE4*	16				DNA RRR
111	YNL088W	*TOP2*	34		2		DNA CM

^a^Percent value of the whole duration of the cell cycle taken from [20,45]. ^b^References for publications where Mbp1 binding was detected. ^c^Number of motifs in the first 200 bp upstream of the TSS (motifs beyond the first 200 bp upstream). ^d^References for publications where the gene was predicted as target of MBF. ^e^DNA RRR, DNA replication, recombination and repair; BP, budding/polarity; SPB, spindle pole body; DNA CM, DNA conformation modification. The top ten predicted targets and all those specific (not detected in [22] or [23]) to our classification are shown. The full list is available in Additional file 3. ND, not determined.

**Table 2:** Summary of targets controlled by SBF

Ranking	Systematic name	Standard name	Cell cycle peak^a^	TF binding^b^	Motifs CRCGAA^c^	Previous classifications^d^	Functional class^e^
1	YER001W	*MNN1*	29	[16-18]	1 (1)	[22,23,36-38]	CW Gly
2	YNL300W	*TOS6*	30	[15-18]	4	[22,23,35-38]	Unknown
3	YKR013W	*PRY2*	25	[16-18]	4	[22,23,35,36,38]	Unknown
4	YOL007C	*CSI2*	24	[15]	4	[22,36]	CW Gly
5	YPL163C	*SVS1*	28	[15-18]	3 (1)	[22,23,35-38]	Others
6	YPL256C	*CLN2*	23	[15]	2 (2)	[23,35,36]	DNA RRR/BP
7	YDR297W	*SUR2*	30	[15]	1	[23]	Others
8	YMR307W	*GAS1*	36	[15-18]	2 (1)	[22,23,36-38]	CW Gly
9	YDR507C	*GIN4*	21	[15-18]	1	[23,37,38]	BP
10	YLR183C	*TOS4*	23	[15]	2 (1)	[22,36]	Others
16	YOL019W		22	[15,17]	1	[36-38]	Unknown
17	YGR140W	*CBF2*	34	[15]	1 (2)		Cell cycle
18	YNL031C	*HHT2*	37	[15]	2	[36]	DNA CM
32	YJL173C	*RFA3*	31		1 (1)		DNA RRR
39	YMR144W		33	[16-18]	1	[35-38]	Unknown
49	YMR179W	*SPT21*	14	[15-18]	1	[35-38]	Others
51	YPL267W	*ACM1*	16	[17,18]	1	[35-38]	Unknown
54	YLR121C	*YPS3*	17	[15]	2	[36]	Others
57	YNL278W	*CAF120*	nd	[15]	2		Others
61	YHR154W	*RTT107*	24		1	[36]	DNA RRR
63	YMR304C-A		nd	[16-18]	0		Unknown
65	YHR173C		36		1		Unknown
67	YBL009W	*ALK2*	36		2		Others
69	YJL080C	*SCP160*	33		2		Cell cycle
70	YKR090W	*PXL1*	27		1		BP
71	YGL093W	*SPC105*	34		1		Cytoskeleton
72	YKL113C	*RAD27*	20		1	[36]	DNA RRR
73	YBR088C	*POL30*	20		1	[36]	DNA RRR
76	YGL225W	*VRG4*	38		2 (1)		CW Gly
77	YDR113C	*PDS1*	33		0		Cell cycle
79	YLR383W	*SMC6*	24		1		DNA RRR
81	YGL012W	*ERG4*	42	[15]	1		Others
84	YPL032C	*SVL3*	40		1		BP
85	YHR050W	*SMF2*	nd		1 (1)		Others
86	YKL049C	*CSE4*	40		1		Cell cycle
87	YBR252W	*DUT1*	40		1		Others
88	YOR099W	*KTR1*	34		1 (1)		CW Gly
89	YLL021W	*SPA2*	31		0 (1)		BP
91	YNL102W	*POL1*	20		0 (1)	[36]	DNA RRR
92	YJR144W	*MGM101*	nd		3 (1)		DNA RRR
93	YLR045C	*STU2*	42		3 (1)	[36]	SPB
94	YAL007C	*ERP2*	37		1		Others

^a^Percent value of the whole duration of the cell cycle taken from [20,45]. ^b^References for publications where Swi4 binding was detected. ^c^Number of motifs in the first 400 bp upstream of the TSS (motifs beyond the first 400 bp upstream). ^d^References for publications where the gene was predicted as target of SBF. ^e^CW Gly, cell wall/glycosylation; DNA RRR, DNA replication, recombination and repair; BP, budding/polarity; SPB, spindle pole body; DNA CM, DNA conformation modification. The top ten predicted targets and all those specific (not detected in [22] or [23]) to our classification are shown. The full list is available in Additional file 3. ND, not determined.

We (and others) find most targets of MBF or SBF to be CCR, with peak expression at the G1 or S phases of the cell cycle (more on this below). However, there are 172 CCR genes with maximal expression in this same cell cycle window that we have not classified as MBF or SBF targets. These are good candidates as false negatives in our analysis. However, only 28 out of these 172 CCR genes are predicted as MBF or SBF targets in at least two previous classifications [22,23,35-38]. Hence, most (approximately 80%) of these targets are likely true negatives. Among those predicted by others, some were in our list below the defined cutoff but close to it (for example, in the MBF list, *KCC4 *was ranked 132, *POL2 *126, and *PLM2 *113; in the SBF list, *HHT1 *was 106). Still, some other genes may have escaped detection because their expression may depend on *BCK2*, which was absent in our experiments. Some candidates within this group are *HLR1*, *FKS1*, and *ELO1 *[27].

We further compared our targets with those provided by Beyer *et al. *[22] and by Holloway *et al. *[23] (Figure 3b). About 70% of our predicted targets were also in the lists of Beyer *et al. *or Holloway *et al. *This was not unexpected since our control sets were based on these studies. By contrast, we only detected 23% of the targets predicted by Beyer *et al. *and approximately 34% of those by Holloway *et al. *Because our study has focused only on those targets that respond in a timely way to Cln3 overexpression in the absence of Bck2, genes that require this protein for their expression would not have been selected. Moreover, some targets controlled by MBF or SBF may also respond to stress, and they would have been likely removed during our gene selection procedure. We examined our expression data for targets solely detected by Beyer *et al. *or Holloway *et al*., and found some 70 genes responding to stress, induced by Bck2 [27], or otherwise selected within our 445 candidates but unsupported as targets by our integrative analysis. However, most targets predicted only by Beyer *et al. *or by Holloway *et al. *would remain unaccounted for under these considerations. It is clear that our study is rather restrictive and that a few true targets of MBF and SBF may be missing from our lists. Also, under different growth conditions MBF and SBF may show distinct binding specificity, which may have been accounted for by these other studies. By contrast, we have predicted 32 targets (29%) of MBF (Table 1) and 32 (34%) of SBF (Table 2) that Beyer *et al. *and Holloway *et al. *failed to detect. Because we have used expression data collected in *swi4Δ *and *mbp1Δ *backgrounds, which surely are more informative about SBF and MBF regulation than expression datasets used in previous studies, our work may provide higher sensitivity (for our experimental conditions) in detecting targets that may have escaped other studies broader in scope.

### Cell cycle behavior

**Figure 4:**
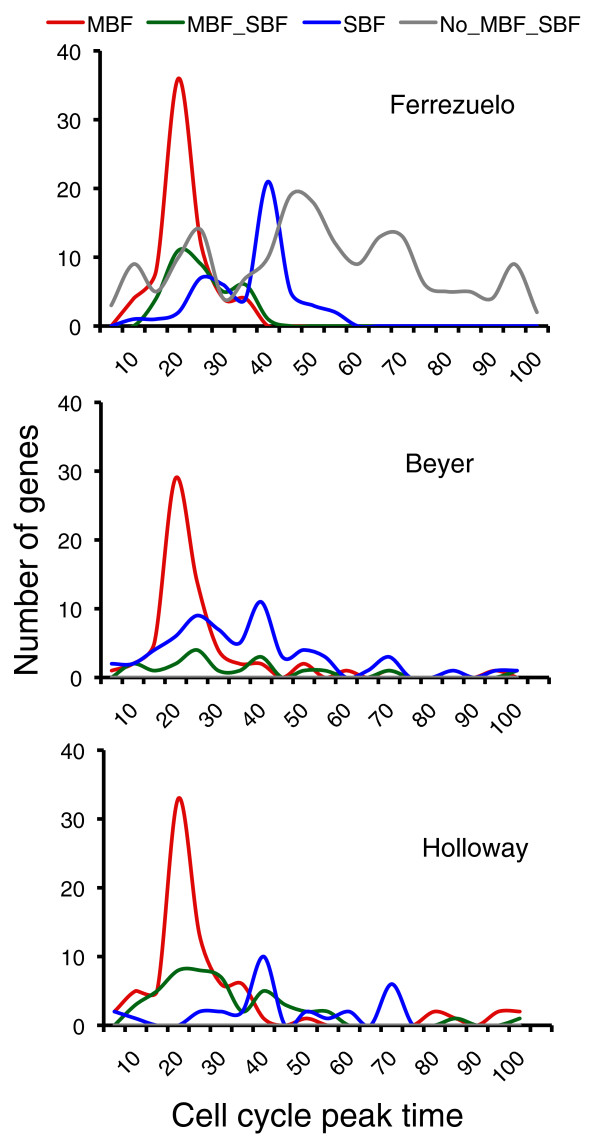
**Cell cycle distribution of targets**. Predicted targets were binned according to their expression peak in the mitotic cell cycle [20,45]. Values on the x-axis are percentages of the whole duration of the cycle, as defined in [20]. Beyer *et al*.'s [22] and Holloway *et al*.'s [23] predicted targets are also shown for comparison. MBF_SBF denotes targets controlled by both TFs; No_MBF_SBF refers to genes from our 445 candidates not classified as MBF or SBF targets.

MBF and SBF are TFs that play a central role during the cell cycle. Hence, we first wanted to visualize the distribution of the expression peaks of their targets throughout the cell cycle (Figure 4). Most targets (92%) were CCR. In comparison, some previous predictions [22,23,35,37] produced a much greater proportion of non-CCR targets. Because we worked with synchronized cultures, explicitly enriched for CCR genes during selection, and used cell cycle regulatory data in our model, this was hardly surprising. MBF targets distributed narrowly, and centered at a time point corresponding to 20% of the whole duration of the cell cycle. Almost identical distributions were observed in previous approaches (Figure 4; Additional file 4). By contrast, the distribution of SBF targets was more variable across studies. In our case, we observed a bimodal distribution (also apparent with Beyer *et al*.'s data) with some SBF targets peaking slightly later than MBF-regulated genes, but most peaking much later (40% point), and few extending beyond 45% of the cycle duration. Significant numbers of SBF targets in other studies [22,23,35,36,38] showed cell cycle peaks beyond this point (Figure 4; Additional file 4). These might be targets for which SBF acts as repressor rather than as activator or which are not controlled by Cln3. Although many SBF targets peak much later than genes regulated by MBF, they are actually activated concurrently or just slightly later [39] (Additional file 5). SBF targets are, however, deactivated much later than MBF targets [39] (Additional file 6). This differential timing of expression of MBF and SBF targets throughout the cell cycle was also apparent in our microarrays, with SBF targets being induced somewhat later and longer than MBF targets. Most likely, this is the consequence of Nrm1-specific repression of MBF targets [33], and Clb2-dependent repression of SBF targets [9,40].

### Experimental validation by ChIP

**Figure 5:**
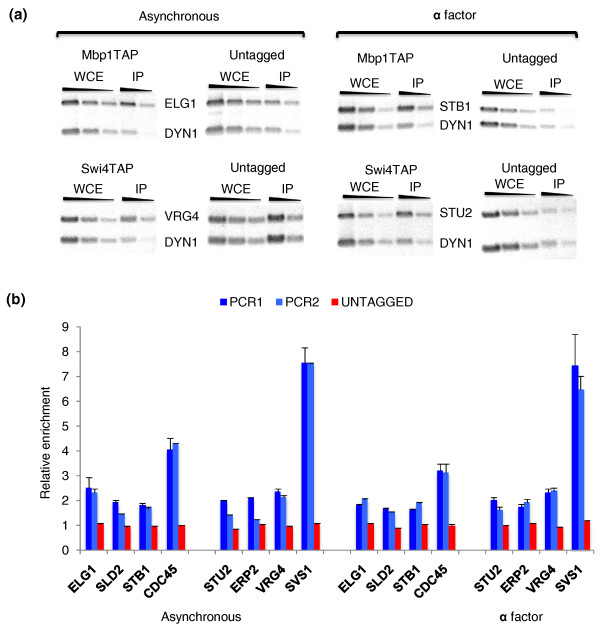
**Experimental validation of predicted targets**. ChIP assays with Mbp1TAP and Swi4TAP were carried out for a number of targets for which TF binding had not been detected before. **(a) **PCR products for predicted targets *ELG1*, *STB1*, *VRG4 *and *STU2 *are shown. Cells were grown either asynchronously or enriched in G1 with α factor. Three dilutions (1:1,500, 1:4,500, 1:13,500 for tagged strains; 1:2,500, 1:7,500, 1:22,500 for untagged strains) of the whole cell extract (WCE) and two (1:5, 1:15) of the immunoprecipitates (IP) were used. PCR was carried out for 28 or 30 cycles for tagged and untagged strains, respectively. As an internal control for non-specificity the gene *DYN1 *was used. The PCR product amplified from this gene was several kilobases away from the closest promoter. **(b) **Quantification of ChIP assays. Optical density of bands was measured with ImageJ. The relative enrichments shown are calculated as ratios of specific to non-specific (*DYN1*) products in the IP compared to the input (WCE). Two independent PCRs were carried out per gene tested (just one PCR in the untagged strains). The average and standard deviations (error bars) of two or three different exposures are shown. Genes *ELG1*, *SLD2*, *STB1 *and *CDC45 *(positive control) were tested in the Mbp1TAP ChIP; genes *STU2*, *ERP2*, *VRG4 *and *SVS1 *(positive control) were tested in the Swi4TAP ChIP.

To validate experimentally our predictions, we performed ChIP assays. For each TF, we chose three targets for which binding had not been detected previously. *ELG1*, *SLD2*, and *STB1 *(ranked 56, 69 and 106, respectively) were chosen as MBF targets, and *VRG4*, *STU2*, and *ERP2 *(ranked 76, 93 and 94, respectively) as SBF targets. Only *STU2 *was predicted as a SBF target by just one previous analysis [36]. As positive controls we chose *CDC45 *and *SVS1 *for MBF and SBF binding, respectively. Both genes bound these TFs in previous genome-wide location analyses [15-18], and are predicted as targets by all previous classifications [22,23,35-38]. *CDC45 *had two ACGCG motifs (Mbp1 binding site) in the first 200 bp upstream of the transcription start site (TSS), whereas the three MBF targets tested contained just one each. *SVS1 *and *STU2 *had three CRCGAA motifs (Swi4 binding site) in the first 400 bp upstream of the TSS, *VRG4 *contained two, and *ERP2 *only one. We designed PCR primers targeting these regions. As control for non-specificity we chose a fragment of the coding sequence of *DYN1*. This gene is one of the largest in the *S. cerevisiae *genome, and thus this region is more than 6 kb away from the closest promoter. In addition, we carried out parallel ChIPs with an untagged strain. As source material for the ChIPs, we used both asynchronous cultures and G1-enriched cultures by treatment with α factor. Somewhat unexpectedly, however, G1 enrichment did not improve detection of MBF or SBF binding. On the contrary, our results are quite comparable irrespective of the growth conditions (Figure 5). Importantly, these constitute two independent ChIP experiments.

We found specific enrichment for all the genes tested when compared to the non-specific control *DYN1 *(Figure 5). As expected, the relative enrichments for the untagged strain were close to one for all the genes and conditions. The positive controls, *CDC45 *and *SVS1*, showed approximately 4-fold and 7-fold enrichments, respectively, whereas our test targets gave values in the range of 1.5 to 2. *STU2 *and *ERP2 *gave the greatest variability, but considering both experiments and all the PCRs performed, we also conclude that there is some enrichment for these genes. These are particularly noteworthy because they are ranked last in our list of SBF targets. Although the enrichments for test genes may seem modest, particularly when compared to that for *SVS1*, this result was anticipated because higher values would have been unlikely to escape detection in genome-wide location analyses.

### Validation by functional enrichment

**Figure 6:**
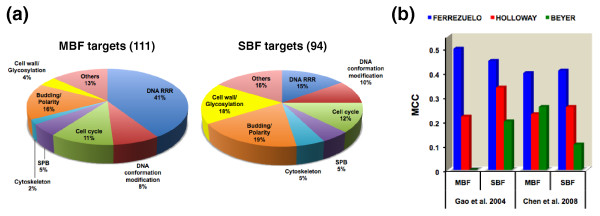
**Quality assessment of our predictions**. **(a) **Functional classification of predicted targets. Functional classes are based on the MIPS functional catalog, but sometimes we merged several classes, and they were adapted to make them virtually non-overlapping. DNA RRR, DNA replication, recombination and repair; SPB, spindle pole body. Thirteen MBF and 14 SBF targets were of unknown function; they are not considered in the percent calculation. **(b) **Comparison of the predictive power of our classification with those of Beyer *et al. *[22] and Holloway *et al. *[23]. MCC was used to assess the ability of each classification to detect true regulatory TF-target associations in the case of divergently transcribed genes for which binding had been reported (Gao *et al. *[37]; Chen *et al. *[44]).

To further validate our predictions, we analyzed the biological functions of our targets (Figure 6a). Because no functional annotation was used at any step in our TF-target assignment approach, gene functions provide an independent quality assessment of our predictions. It has been previously proposed that MBF and SBF control genes with distinct and dedicated roles. Thus, many MBF targets would be involved in DNA replication, repair and DNA processing in general, whereas many SBF-controlled targets seem to be involved in membrane and cell wall biogenesis [15,41,42]. In agreement with this, we have found statistically significant enrichment (*P *‹ 10^-15^) in genes involved in DNA replication, repair and recombination among our MBF targets. We also found significant enrichment (*P *‹ 2 × 10^-4^) for SBF-regulated targets involved in cell wall biogenesis and integrity, as well as protein glycosylation. We considered these two functional classes together because many cell wall components are highly glycosylated proteins, and cell wall integrity thus strongly depends on protein glycosylation (reviewed in [43]). We next examined the functional consistency of our classification by comparing the distribution in different functional classes of unique versus shared targets, taking as reference the lists provided by Beyer *et al. *[22] and Holloway *et al. *[23]. We found no statistically significant differences (two-tailed Fisher exact test, *P *‹ 0.05) between these two sets in any of the functional categories considered. By comparison, a similar analysis performed with Beyer *et al*.'s and Holloway *et al*.'s classifications showed significantly fewer genes dedicated to DNA replication, recombination and repair among their unique MBF targets than in those shared with other classifications (*P *‹ 2 × 10^-5 ^and *P *‹ 2 × 10^-3^, respectively). Beyer *et al*.'s SBF targets were lacking in cell cycle genes (*P *‹ 0.01) and those involved in cell wall and glycosylation (*P *‹ 6 × 10^-5^). By contrast, Holloway *et al*.'s specific MBF targets included more genes involved in cell wall and glycosylation (*P *‹ 0.02). In conclusion, our classification shows higher functional internal consistency than the predictions from these previous studies. This consistency reinforces the idea that we have been able to find many real targets that have escaped previous analyses.

### Evaluation of predictive power: the case of divergently transcribed genes

Divergently transcribed genes offer another approach to evaluate the quality of our predictions. These genes share their promoter regions, and because in yeast intergenic regions are usually short, ChIP-chip data alone cannot distinguish whether both or only one gene (or none) may be regulated by the bound TF. Several studies [37,44] have integrated expression data together with ChIP-chip data to establish which divergent genes are likely or unlikely to be regulated by bound TFs. These works provide independent predictions that can be used as benchmarks to compare the predictive power of other classifications. Compared to the experimental data we have used, Beyer *et al*.'s and Holloway *et al*.'s analyses have arguably used datasets more akin to those used previously [37,44]. Despite this, our classification outperformed both Beyer *et al*.'s and Holloway *et al*.'s in predicting true regulation in divergently transcribed genes as measured by MCC (Figure 6b). These other classifications displayed much lower specificity and precision, similar accuracy, and higher sensitivity than ours (data not shown). The greatly diminished specificity (higher number of false positives) of these classifications may be explained by the fact that both seem to rely strongly on genome-wide binding data.

### Internal consistency: distribution of motifs in MBF targets

**Figure 7:**
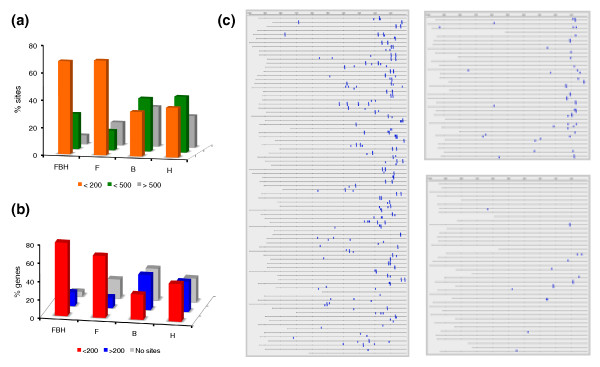
**Mbp1 binding motif distributions at gene promoters**. **(a) **Proportion of ACGCG sites located within the first 200 bp, from 200 to 500 bp, and beyond 500 bp at the promoters of MBF targets that are specific to this work (F), to Beyer *et al. *[22] (B), to Holloway *et al. *[23] (H), or that are common to all three studies (FBH). **(b) **Proportion of MBF targets with ACGCG sites within the first 200 bp upstream of the TSS, beyond 200 bp, or without such sites; FBH, F, B, and H as before. **(c) **Promoter representations with the location of ACGCG sites (blue). Left panel, MBF targets in our work shared with the aforementioned studies. Right top panel: our specific MBF targets. Right bottom panel: random set of genes with ranking values from 200 to 445 in our MBF classification. Every line represents a gene promoter from the TSS (right end) up to -1,000 bp upstream of the START codon. For all analyses in this figure, scores were recalculated without the motif classifier.

The MBF targets used as positive control in our analysis were highly enriched for Mbp1 binding motifs (ACGCG) located proximal (‹200 bp) to the TSS. Whereas 65% of these targets had at least one binding site in the first 200 bp upstream of the TSS, only 4.5% of genes in our negative control did. Similarly, the SBF control genes were enriched in Swi4 binding motifs (CRCGAA), but they were neither so narrowly distributed upstream of the TSS nor so highly enriched (78% versus 33%). Strikingly, even when we recalculated the scores without the motif classifier - hence, no information concerning sequence motifs was used - the vast majority of the MBF targets still presented the ACGCG motif in their promoters with a clearly biased distribution towards the proximity of the TSS (Figure 7). This was true irrespective of whether the predicted targets were common to other studies or unique to our work. By contrast, a random set of non-MBF targets did not show this pattern (Figure 7c). We next examined the distribution of motifs in the promoters of the MBF targets predicted by Beyer *et al. *and Holloway *et al. *We considered four groups of targets: those detected in all three studies and those unique to only one study. Because the classifications by Beyer *et al. *and Holloway *et al. *included motif information, we expected to find enrichment of MBF binding motifs. Indeed this was the case, but these motifs were much more scattered along the full length of promoters in Beyer *et al*.'s or Holloway *et al*.'s targets than in the common set or in our specific targets (Figure 7a). Consequently, the proportion of genes containing sites in the first 200 bp upstream of the TSS in the common set and in our specific group was greater than in the specific sets of the other two studies considered (Figure 7b). Hence, this analysis strongly suggests that our MBF targets constitute a more homogeneous group than those previously described [22,23]. Previous analyses may have detected condition-specific targets of Mbp1 that we may have missed under our more restrictive experimental investigation. Should this be the case, however, the distinct distribution of motifs would suggest that positional information at promoters may play a role in the response to one or another cellular cue.

### Evaluation of genome-wide location datasets

**Figure 8:**
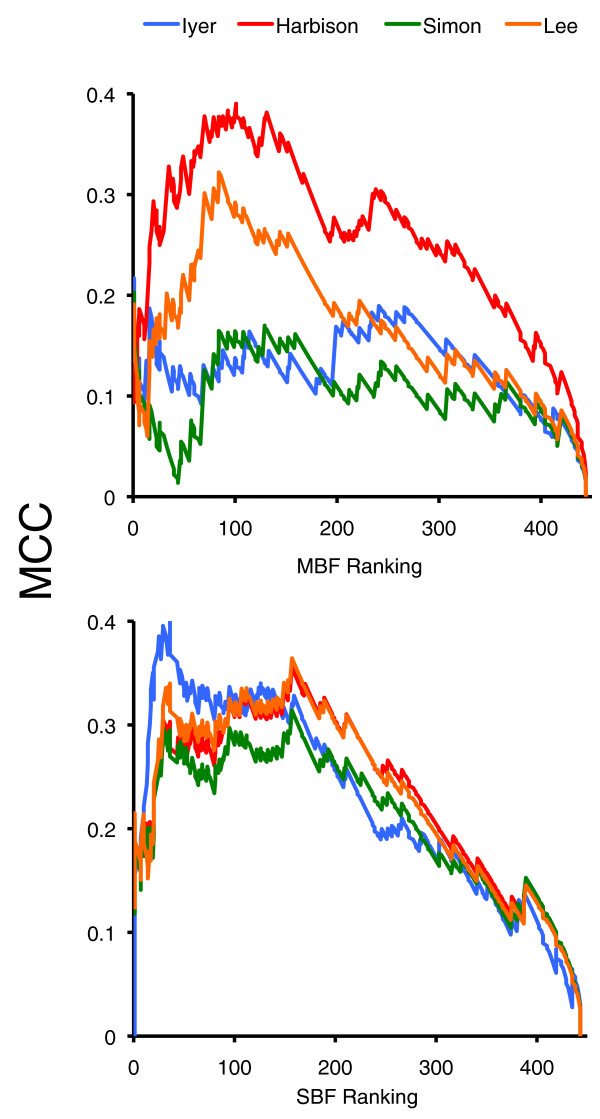
**Quality assessment of location analyses**. The predictive power (MCC) of different location analyses was evaluated with our classifications as benchmarks. MCC values are represented throughout our ranked list of candidates. Work by Iyer *et al. *[15], Simon *et al. *[16], Lee *et al. *[17], and Harbison *et al. *[18] were considered. For these analyses, we did not include explicit binding information in our classifications.

Finally, we used our classification as a benchmark to compare the predictive value of the different genome-wide location analyses involving Mbp1 and Swi4. To this purpose, we produced classifications leaving the binding information classifier out. Note that the datasets generated by Young and co-workers [16-18] were used by Beyer *et al. *and Holloway *et al. *in their analyses, and because our control sets were derived from those studies, our predictions cannot be considered fully independent from those datasets. We used MCC to assess the predictive power of these datasets. For Mbp1, regardless of the cutoff chosen in our classification, Harbison *et al*.'s [18] data greatly outperformed the others (Figure 8), especially those by Simon *et al. *[16] and Iyer *et al. *[15]. This may stem from the fact that Harbison *et al. *performed their Mbp1 ChIPs under several growth conditions, providing a considerably larger number of targets. In fact, whereas the accuracy and specificity of all four studies analyzed were similar, Harbison *et al*.'s dataset was significantly more sensitive than the others (data not shown). For Swi4, Iyer *et al*.'s dataset slightly outperformed the other three studies, at least for a cutoff of 100 or lower, which is a reasonable threshold for SBF-regulated genes in our classification (Figure 8). This difference was underscored by the fact that, contrary to the others, Iyer *et al*.'s study provided a dataset that was fully independent of our classification.

## Discussion

The transcriptional program at START is driven by the related TFs MBF and SBF. Cln3 is the most upstream activator of START. It functions by activating the CDK Cdc28, which then inhibits repressors of SBF and MBF, leading to the activation of their target genes [12,13]. Cln3 is not, however, the only activator operating at START. For instance, it shares an essential function with Bck2 of promoting the G1 to S transition of the cell cycle [24-26], and we have recently shown that Bck2, at least when overexpressed, induces many genes at this point [27]. Here we provide an extensive list of genes that are activated by Cln3 in the absence of Bck2 in an MBF- or SBF-dependent manner. In fact, it is likely that Cln3 functions solely, at least as a transcriptional activator, through MBF and SBF because all known functions of Cln3 depend on Swi6 [27,28], overexpression of Cln3 at cell cycle stages other than G1 has little effect on gene activation [27], and here we have shown that Cln3 is unable to induce any of its targets in a *swi4Δ mbp1Δ *background.

We produced our list of Cln3 targets in two steps. First, we generated new genome-wide experimental data that are arguably more informative for this purpose than other datasets available in the literature. This is so because we studied the effects on gene expression of overexpressing Cln3 in synchronized cultures, and most importantly because we used a battery of deletion strains lacking components of MBF and/or SBF. Second, because Cln3 needs MBF or SBF to promote gene expression, we integrated our data together with other published datasets to determine the targets of Mbp1 and Swi4. This has allowed us to distinguish direct targets of Cln3 from genes induced indirectly as a result of cell cycle progression in our experiments. It is possible, however, that some of the genes regulated by Mbp1 or Swi4 are not direct targets of Cln3. Cln1 and Cln2 are involved in a positive feedback mechanism promoting transcriptional activation at START [14]. Hence, it is unclear whether the induction we see is solely due to overexpressed Cln3, or most likely to Cln1, Cln2 and Cln3 acting in concert. Interestingly, most MBF targets seem to be insensitive to overexpressed Cln1 (our unpublished results).

Following previous approaches [22,30], we have developed a single probabilistic model based on Bayesian statistics that allows the integration of data from heterogeneous sources. Integration is important because with expression data alone it is difficult to distinguish direct from indirect regulation as well as compensating mechanisms of redundant factors, whereas TF binding or motifs at promoters lack functional information. From our experiments, we have made available to our model expression data concerning the time and extent of induction, and how these are affected in deletion mutants. From others, we have taken information on TF binding, Cln3 induction (under non-progressive conditions), Clb2 repression, and cell cycle behavior [3,15-18,20,45]. We have also integrated information about binding motifs at promoters. Doubtless, the dominant feature in our classification is gene expression. This is, however, rather specific and more informative than expression datasets typically used in genome-wide studies on transcriptional networks. In general, it seems these studies give more weight to ChIP-chip data (see, for example, Beyer *et al. *[22] and Holloway *et al. *[23]).

We have validated our predictions in two ways. First, and most important, we have demonstrated by ChIP assays that Mbp1 and Swi4 bind the promoters of predicted targets for which binding had not been detected before [15-18]. Second, our predictions show high enrichment in biological functions previously attributed to MBF or SBF [15,41,42]. Importantly, and contrary to other analyses [22,23], this was true also for the set of targets that was specific to this study, indicating that our classification maintains internal functional consistency. On the other hand, our classification shows greater predictive power than previous ones [22,23] as tested by their ability to discriminate regulatory targets between divergently transcribed genes.

We have used our TF-target assignments as a benchmark to assess the quality of several genome-wide TF binding datasets [15-18]. Our analysis suggests that whereas for Mbp1 the study by Harbison *et al. *[18] is superior to the others, for Swi4 Iyer *et al. *[15] is the best performer. Interestingly, Harbison *et al. *provided a more thorough study of Mbp1 (several conditions assayed) than of Swi4, and conversely Iyer *et al. *performed many more ChIP-chip experiments for Swi4 than for Mbp1. It is likely, then, that more experimental ChIP-chip data may considerably improve the quality of available datasets.

Our predicted MBF targets are highly enriched in ACGCG sequences. Strikingly, the position of this motif is strongly biased towards the first 200 bp from the TSS. Importantly, these features remain unchanged even when the motif information classifier is not incorporated into our model. Hence, this constitutes another independent confirmation that our classification must have captured biologically meaningful predictions. By contrast, this promoter architecture is not maintained in most Mbp1 targets specific to other models [22,23]. It is possible that association of Mbp1 with partners other than Swi6 may change its binding specificity. SBF targets show enrichment of CRCGAA sequences, but their more scattered distribution suggests that SBF-controlled promoters are more complex than MBF-regulated promoters. In agreement with this, combinatorial regulation involving Swi4 and other factors seems commonplace [22,23,46].

The apparently simpler architecture of MBF target promoters correlates with a narrow distribution in their expression peak during the mitotic cell cycle. By contrast, SBF targets show a more spread bimodal distribution. This may likely be due to combinatorial regulation with Ste12 and forkhead TFs [22,23,46]. The bulk of SBF targets peaks much later than genes regulated by MBF. This is so mainly owing to their different inactivation timing, and not so much because SBF targets are activated much later. In fact, most SBF targets are activated just slightly later. MBF-regulated genes are subject to specific repression by Nrm1 [33], a G1/S cell cycle-regulated gene, as cells proceed from G1 to S phase, and before Clb/CDK activity raises. By contrast, SBF is repressed only later, when Clb2 is expressed and its activity is high [9,40]. Hence, the set of targets we have predicted here recapitulate known cell cycle regulatory mechanisms.

It has been controversial whether Whi5 represses only SBF [13] or both SBF and MBF [12]. Recently, the role of Stb1 as an activator and repressor of both SBF and MBF has also been proposed [47-50]. Here, we have predicted *STB1 *as a target of MBF, and we have demonstrated Mbp1 binding to the *STB1 *promoter by ChIP assays. This raises the possibility of Stb1 being involved in feedback mechanisms as well as linking MBF and SBF regulation at START. Nonetheless, the small but appreciable delay in the activation of most SBF targets as compared to MBF-regulated genes, whether related to Stb1 function or not, supports the existence of different activating mechanisms for these TFs.

## Conclusions

Here we have provided the transcriptional network activated by the cell cycle regulator Cln3 through the TFs SBF and MBF. We have validated our TF-target predictions both experimentally by means of ChIP assays, and computationally by studying the functional enrichment of target genes. Although likely still incomplete, our network appears to be more accurate (higher predictive power and internal consistency) than others previously proposed. Likely, this stems from the integration of new experimental data with other available genome-wide datasets, and from relying less on TF binding studies than other previous integrative models. We believe our work exemplifies the need to generate more informative experimental data to build detailed and reliable networks. This work and similar approaches may be keystones to the development of accurate computational models of the cell cycle.

## Materials and methods

Strains used in the expression profiling experiments were MATa haploid W303 derivatives. Their relevant genotypes are shown in Figure 1. General procedures for the construction of strains, growth conditions, budding count, DNA content analysis, RNA isolation as well as microarray hybridizations and data analysis have been described previously [27]. Microarray data have been deposited in ArrayExpress under accession number [ArrayExpress:E-TABM-764].

### Gene selection

To select for genes specifically induced by Cln3 or by cell cycle progression, we used five slightly different criteria based on gene clustering [51]. Two selection methods used visual inspection only. One has been described previously [27]. The other was similar except that only the strains used in this work, but not the P_*GAL1*_·*BCK2 *strains used in our previous study, were used. Another method used first a visual selection and then a second selection based on cell cycle enrichment. Two other methods were based solely on cell cycle enrichment, but for one we first filtered out inconsistent expression between duplicate experiments evaluated in the P_*GAL1*_·*CLN3 bck2Δ *strain. Throughout this study we consider CCR genes as those belonging to a consensus list of 648 cell cycle genes (Additional file 7) that appear among the top 800 ranked in at least three of five cell cycle studies [3,20,45,52,53].

### Probabilistic model

We have followed others' ideas [22,30] to develop a Bayesian probabilistic model. We have used a unified scoring scheme that received input from nine different classifiers (see below). Most classifiers were binned into four mutually exclusive groups. To delimit each group, we chose three random sets of 40 elements from our list of 445 genes (see Results). The 40 elements in each set were sorted by their values within each classifier, and the 10th, 20th, and 30th ranked values in each random set were averaged, respectively. These average values were used as thresholds to delimit the bins. Each bin was then assigned a weight calculated as a log likelihood score (LLS):

LLS = ln(P(bin_i_/positive)/P(bin_i_/negative))

where P(bin_i_/positive) and P(bin_i_/negative) are the frequencies of positives and negatives from control sets (see below), respectively, that belong in bin i. The total LLS for each gene in our list was the result of adding all individual LLSs from the corresponding bins for the nine classifiers considered. All scores can be found in Additional file 2.

### Control sets

To train our model, we created positive and negative control sets for both factors, Mbp1 and Swi4. Positive and negative interactors were chosen from our list of 445 candidates. Positives were genes defined as targets of Mbp1 or Swi4 in both Beyer *et al. *[22] and Holloway *et al. *[23]. We avoided picking up genes regulated by both Mbp1 and Swi4, as well as other cell cycle TFs (Ste12, Fkh2, Ndd1 or Mcm1). Because this gave rise to too few positives, especially for Swi4, we added some targets that were top ranked in either classification (although not in both). For these, we also avoided those regulated by both factors. We ended up with 40 positives for Mbp1 (90% shared by Beyer *et al. *and Holloway *et al*.), and 32 positives for Swi4 (50% shared by Beyer *et al. *and Holloway *et al*.). The negative set for Mbp1 (or Swi4) consisted of randomly selected genes from our list of 445 candidates that were not reported to be regulated by Mbp1 (or Swi4) in Beyer *et al*.'s or Holloway *et al*.'s studies. We selected five groups of 40 genes for Mbp1, and five groups of 32 genes for Swi4. The five groups were merged into a single negative set.

### Classifiers

We used nine classifiers integrating different lines of evidence: one from TF binding data, one from TF motifs, four from the expression data we generated in this study, one from expression profiling during the cell cycle, and two from the expression profiling upon Cln3 or Clb2 overexpression, as reported in a previous study [3].

#### Transcription factor binding information

We used TF binding data from four genome-wide studies that used ChIP-chip technology [15-18]. We considered the assignments proposed by Iyer *et al. *[15], and those TF-target interactions with a *P*-value ‹0.001 from the other three studies. For MBF, we evaluated three conditions: none of the studies, only one study, and more than one study detected an interaction. For SBF, we did the same, but SBF interactions detected by Iyer *et al. *were considered more reliable and consequently given more weight. The rationale behind this is that Iyer *et al. *performed multiple ChIP-chip experiments with Swi4, and they arguably produced better quality data for this factor. Also, preliminary comparisons of our expression dataset with that of Iyer *et al. *and from the other three ChIP-chip studies suggested better agreement with the former study.

#### Transcription factor motifs

For MBF, we evaluated whether the promoters of genes had at least one MCB consensus site (ACGCGT) within the first 200 bp upstream of the TSS or not. For SBF, we examined the presence of at least one SCB consensus site (CRCGAA) located within 400 bp of the TSS. The TSS information was obtained from two recent genome-wide studies [54,55].

#### Expression data

We evaluated six classifiers from the expression profiles generated in this study, and three more from data generated by others. (1) The time of peak expression in the wild-type strain. This parameter was divided into four groups according to the sampling performed, that is, 20, 40, 60 and 80 min. (2) The value at 20 minutes in the wild-type strain. (3) The ratio between the maximum value in the wild-type strain series and the maximum in the *mbp1Δ *mutant as well as (4) the correlation between the profiles in the wild-type and in the *mbp1Δ *backgrounds. These two classifiers were used only for Mbp1. For Swi4, we evaluated (5) the average value at 40 and 60 minutes in the wild type as well as (6) the ratio between the maximum value at 20 or 40 minutes in the wild type and the maximum value in the *swi4Δ *background. From the work of Spellman and co-workers [3], we analyzed (7) the value of induction upon Cln3 or (8) upon Clb2 overexpression. Finally, we also considered (9) the time of peak expression during the mitotic cell cycle [20,45].

### Evaluation of predictions and thresholding

We first created several benchmarks of positives and negatives. Positive benchmarks for both Mbp1 and Swi4 were created with 40 genes each. All benchmarks contained ten genes that had been reported as regulated by both factors in previous classifications [22,23]. The remaining 30 genes for each particular benchmark were randomly selected among those targets regulated by Mbp1 (or Swi4) in any of those studies. None of the genes in the benchmark sets had been used before in the training sets. We generated two positive benchmarks for each factor. Negatives for Mbp1 or Swi4 were randomly selected among those genes that were not regulated by Mbp1 or Swi4, respectively, in Beyer *et al*.'s and Holloway *et al*.'s studies. For each factor, we randomly selected 40 genes twice, and merged the two groups. Hence, the negative benchmarks contained somewhat fewer than 80 genes each.

Throughout this study we have used several statistical measures commonly employed to assess the quality of binary classifications. They are defined as follows:

where TP is true positives, TN true negatives, FP false positives, and FN false negatives.

To select thresholds, we calculated these measures at any given position in our classifications. We averaged (geometric mean) the values obtained with each positive benchmark. We chose as cutoff a ranking value that produced high specificity and precision (›80%) as well as a high value for the MCC. Likely, these quality measures produced underestimated values because at least some of the targets in the positive benchmarks may not be true positives (many were reported as targets by Beyer *et al. *or Holloway *et al*., but not by both studies) and some of the genes in the negative benchmarks may actually be positive. In fact, we have predicted some targets that escaped previous detection.

### ChIP assays

Strains used in ChIP assays were derived from BY4741 (*MATa his3Δ1*, *leu2Δ0*, *met15Δ0*, *ura3Δ0*). We tagged Mbp1 or Swi4 with tandem affinity purification (TAP) tag [56]. Correct tagging was checked by PCR and western blotting. Tagged strains and untagged control were grown in YPD at 30°C to an OD_600 _of ‹0.25, split in two, α factor (5 mg/l) was added to one culture, and all cultures were incubated at 30°C for an extra 90 minutes. At this point, in the cultures with α factor most cells were arrested at G1 as determined by microscope inspection. We used 40 ml of culture per ChIP. These were carried out as previously described [49] with modifications. Briefly, after formaldehyde cross-linking, cells were broken in a BioSpec (Bartlesville, OK, USA) mini-beadbeater-16 (6 pulses of 1 minute with 1 minute on ice between pulses), chromatin was sheared in an MSE (London, UK) soniprep-150 sonicator (power 10, 6 pulses of 15 s, ice 1 minute between pulses), and clarified extracts were incubated with 50 μl magnetic beads (Dynabeads Pan mouse IgG, Invitrogen Dynal, Oslo, Norway) for 90 minutes at 4°C. Washes were carried out at room temperature, and after elution and reversal of the cross-link, we treated with proteinase K (0.25 mg/ml, 2 h, 37°C). DNA was purified with a Qiagen (Valencia, CA, USA) column (PCR QIAquick PCR purification kit) and eluted with 100 μl elution buffer (10 mM Tris-Cl pH 8.5). Finally, RNase A was added to 0.5 mg/ml and incubated for 2 h at 37°C. PCR was carried out for 28 (tagged strains) or 30 cycles (untagged controls). PCR products were separated in 2.4% agarose gels, stained with SYBR gold (Invitrogen, Carlsbad, CA, USA), and imaged with an AlphaDigiDoc RT2 gel documentation system (Alpha Innotech, Santa Clara, CA, USA). Quantification of bands was performed using ImageJ.

### Miscellaneous

For our functional analysis, we focused on several functional classes that were more over-represented among our predicted targets according to the Munich Information Center for Protein Sequences (MIPS) functional catalog [57]. Sometimes we removed genes to make them non-overlapping. The final classes considered were as follows: cell wall and glycosylation; budding and polarity; spindle pole body (SPB); cytoskeleton (excluding SPB, budding and polarity members); DNA conformation modification; DNA replication, recombination and repair (excluding members involved in DNA conformation modification); and cell cycle (excluding genes involved in DNA processing, SPB, budding or polarity). The heat map in Figure 2 was generated with the Java TreeView software [58]. Venn diagrams in Figure 3 were created with an Applet from [59]. To match and visualize motifs at promoters we used the tools implemented in the Regulatory Sequence Analysis Tools web site [60].

## Abbreviations

bp: base pair; CCR: cell-cycle regulated; CDK: cyclin-dependent kinase; ChIP: chromatin immunoprecipitation; LLS: log likelihood score; MCC: Matthews correlation coefficient; SPB: spindle pole body; TF: transcription factor; TSS: transcription start site.

## Authors' contributions

FF and BF designed the experiments; FF performed the experiments; NC and MA contributed reagents and experimental assistance; FF and MA analyzed the data; FF wrote the paper.

## Supplementary Material

Additional file 1 **Log2 expression values for the 445 candidate genes selected from our microarray analysis. **This file contains log2 expression values (relative to time 0) for the 445 candidate genes selected from our microarray analysis. There are two sheets labeled 'Average_values' and 'Duplicate_experiments'. The 'Duplicate_experiments' sheet contains the values of two independent experiments (denoted _1 and _2 following the name of strain and time). The 'Average_values' sheet contains the data represented in Figure 2, corresponding to the average values of the two independent experiments mentioned above. Arrays are labeled with the relevant genotype of the strain and the time of sampling. Same color is used for all the arrays obtained with the same strain. The background context for all strains was *bck2Δ *P_*MET3·*_*CLN2*. Except for strain *cln3Δ*, cells also had P_*GAL1*_*·CLN3 *at the endogeneous *CLN3 *locus (wt stands for wild type). Click here for file

Additional file 2 **Log likelihood scores for the 445 candidates analyzed in our study. **Matrix containing the individual values assigned to each gene in all nine classifiers used in our model and the final score obtained (column SUM). Each sheet corresponds to one TF. 'PEAK TIME' evaluates the time of peak expression in the wild-type strain in our experiments. 'Value 20' wt' evaluates the value at 20 minutes in the wild-type strain whereas 'Av. value 40-60 wt' (only Swi4) corresponds to the average value at 40 and 60 minutes in the wild type. In 'Corr. wt/mbp1Δ' we assess the value for the correlation coefficient between the expression patterns in the wild type versus the *mbp1Δ *strain. 'max wt/max mbp1Δ' (only Mbp1) refers to the ratio between the maximum value in the wild-type series (20 to 80 minutes) and the maximum in the *mbp1Δ *mutant. Similarly, 'max wt_20-40/max swi4Δ' makes reference to the ratio between the maximum value at 20 or 40 minutes in the wild type and the maximum value in the *swi4Δ *background. For 'Mbp1 motifs' we evaluated whether the promoters of genes had at least one MCB consensus site (ACGCGT) within the first 200 bp upstream of the TSS or not. For SBF ('Swi4 motifs'), we examined the presence of at least one SCB consensus site (CRCGAA) located within 400 bp of the TSS. In 'Mbp1 binding' we evaluate TF binding data from four genome-wide studies that used ChIP-chip technology [15-18]. We considered the assignments proposed by Iyer *et al. *[15], and those TF-target interactions with a *P*-value ‹0.001 from the other three studies. Three conditions were assessed: none of the studies, only one study, and more than one study detected an interaction. The same applies to 'Swi4 binding' but interactions detected by Iyer *et al. *were considered more reliable and consequently given more weight (see Materials and methods for details). In 'cln3' and 'clb2', we analyzed the value of induction upon Cln3 or upon Clb2 overexpression in [3]. Finally, 'CC peak' assesses the time of peak expression during the mitotic cell cycle. Click here for file

Additional file 3 **Full lists of predicted targets. **This file contains the full lists of predicted targets for both factors, MBF and SBF (two sheets labeled correspondingly). Cell cycle peak was taken from [20,45]. References for publications where binding of TF or predictions of the gene as target have been reported are given in the 'TF binding' and 'Previous classifications' columns, respectively. For MBF, the number of ACGCG motifs in the first 200 bp upstream of the TSS and motifs beyond the first 200 bp (between parentheses) are given. For SBF, the number of CRCGAA motifs in the first 400 bp upstream of the TSS and motifs beyond the first 400 bp (between parentheses) are given. 'SGD description' refers to gene product description in the *Saccharomyces *Genome Database. 'Functional class' includes some modified functional classes according to the MIPS functional catalog (see Materials and methods for details). Click here for file

Additional file 4 **Cell cycle distributions of predicted targets according to the timing of peak expression. **This figure constitutes an expansion of Figure 4 of the paper. It shows the cell cycle distributions of predicted targets according to the timing of peak expression for a number of classifications: F, this study; B, [22]; H, [23]; BJ, [35]; T, [36]; W, [38]; G, [37]. Values on the x-axis are percentages of the whole duration of the cycle, as defined in [20]. Red, MBF targets; blue, SBF targets; green, both MBF and SBF targets; gray, our 445 candidates not classified eventually as MBF or SBF targets. Note that y-axis scales vary across classifications. Click here for file

Additional file 5 **Cell cycle distributions of predicted targets according to the timing of activation of expression from **[39]. Letter and color keys as in Additional file 4. Click here for file

Additional file 6 **Cell cycle distributions of predicted targets according to the timing of deactivation of expression from **[39]. Letter and color keys as in Additional file 4. Click here for file

Additional file 7 **The 648 genes considered as cell cycle regulated in this study. **These genes appear among the top 800 ranked in at least three of five cell cycle studies [3,20,45,52,53]. The time of their peak expression is also shown [20,45]. Click here for file
