# Supplementary figures and images for: The transcriptional network activated by Cln3 cyclin at the G1-to-S transition of the yeast cell cycle
Source: Genome Biol. 2010 Jun 23;11(6):R67. doi: 10.1186/gb-2010-11-6-r67 (PMC2911115; doi:10.1186/gb-2010-11-6-r67)

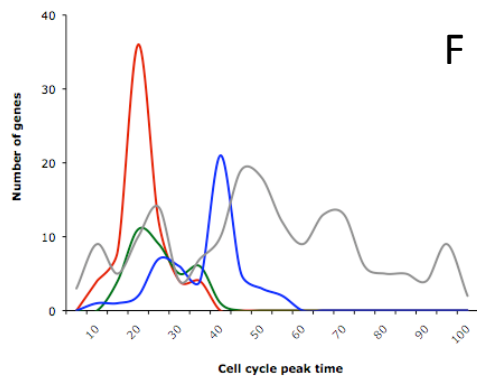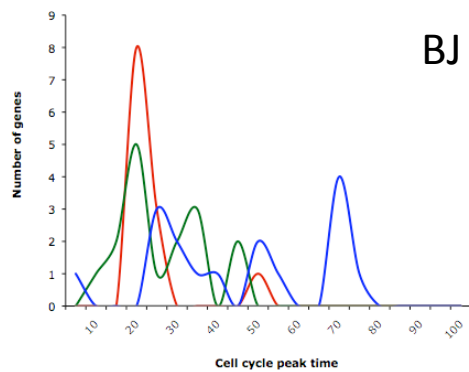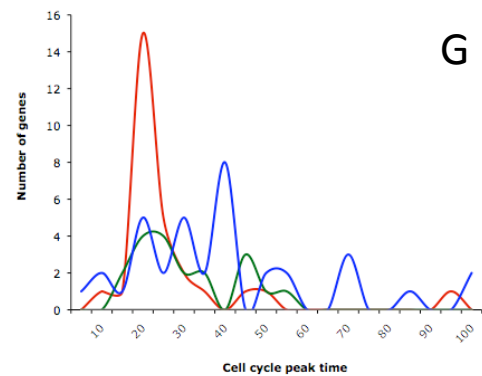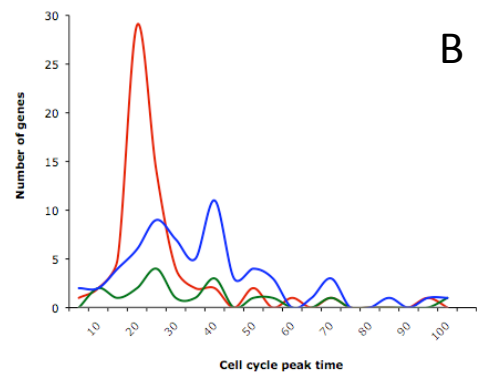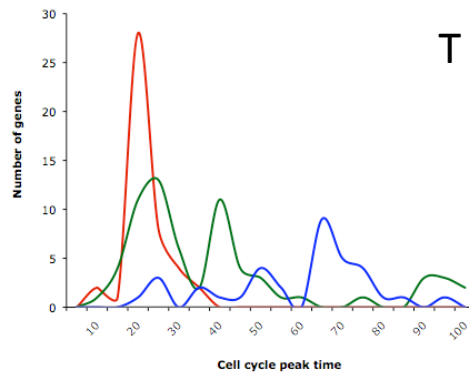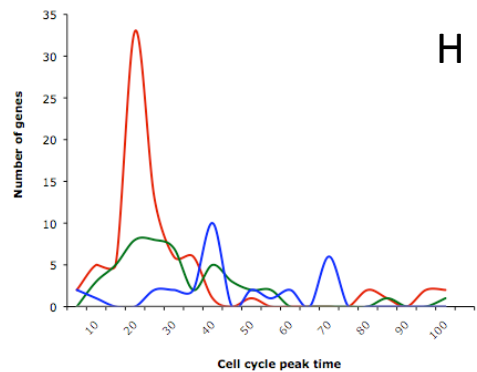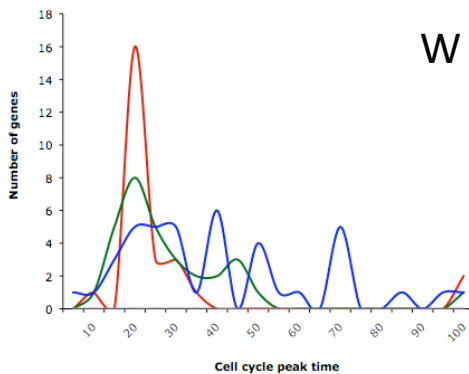

Peak

Supplement: Additional file 4 — Cell cycle distributions of predicted targets according to the timing of peak expression. This figure constitutes an expansion of Figure 4 of the paper. It shows the cell cycle distributions of predicted targets according to the timing of peak expression for a number of classifications: F, this study; B, [22]; H, [23]; BJ, [35]; T, [36]; W, [38]; G, [37]. Values on the x-axis are percentages of the whole duration of the cycle, as defined in [20]. Red, MBF targets; blue, SBF targets; green, both MBF and SBF targets; gray, our 445 candidates not classified eventually as MBF or SBF targets. Note that y-axis scales vary across classifications. [file gb-2010-11-6-r67-S4.PDF]

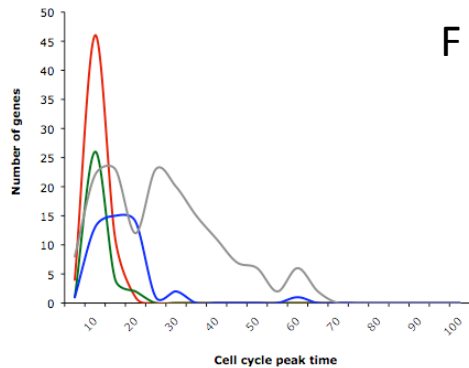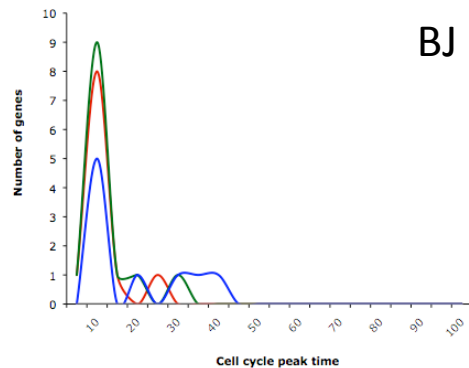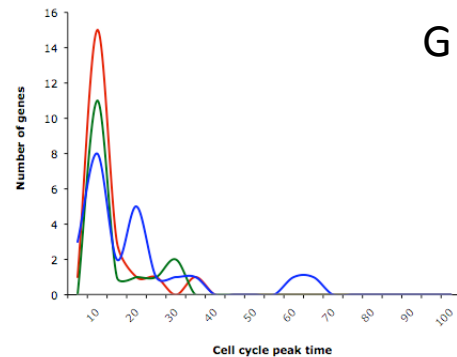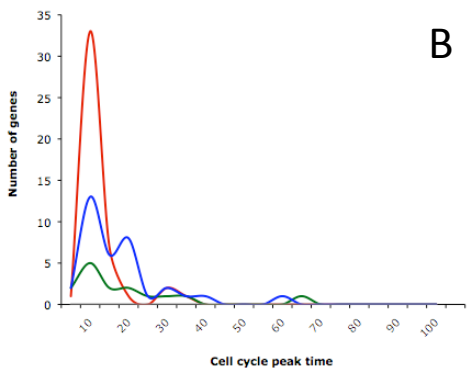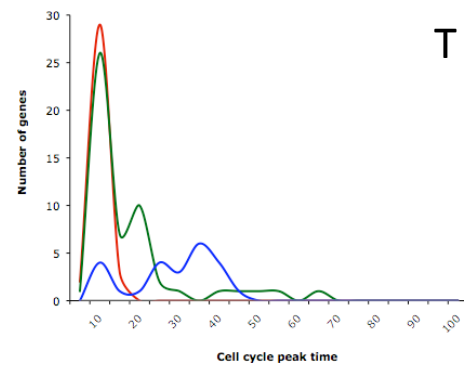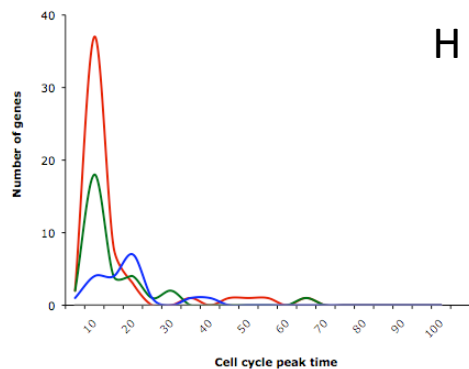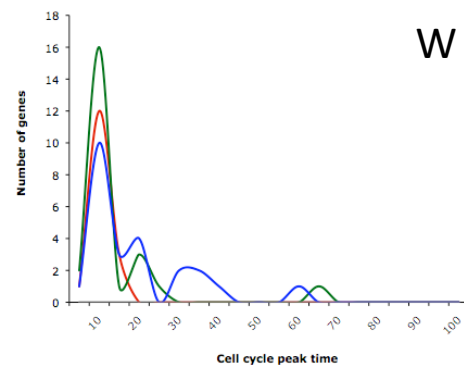

Activation

Supplement: Additional file 5 — Cell cycle distributions of predicted targets according to the timing of activation of expression from [39]. Letter and color keys as in Additional file 4. [file gb-2010-11-6-r67-S5.PDF]

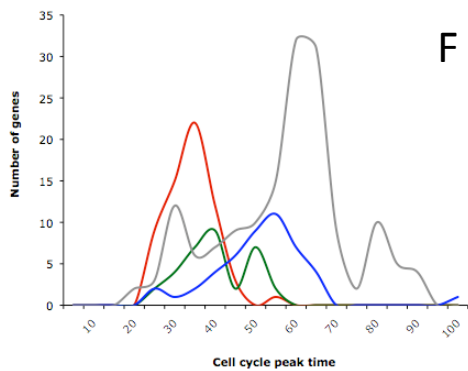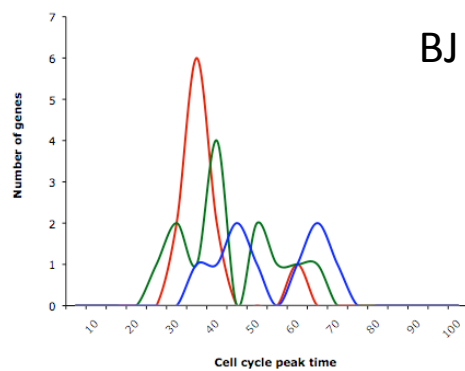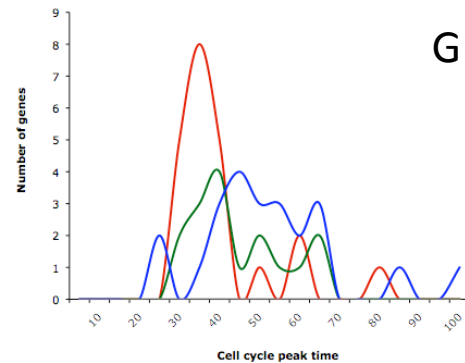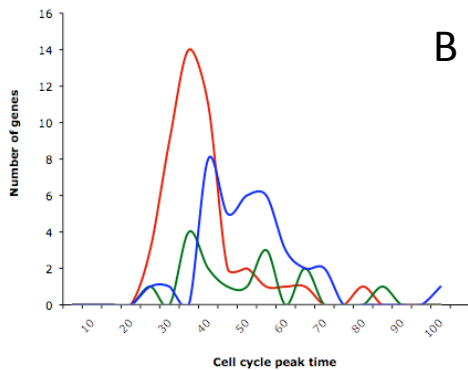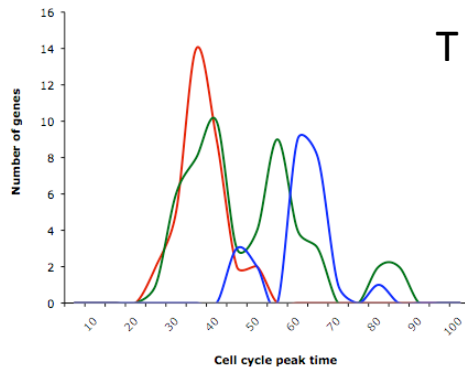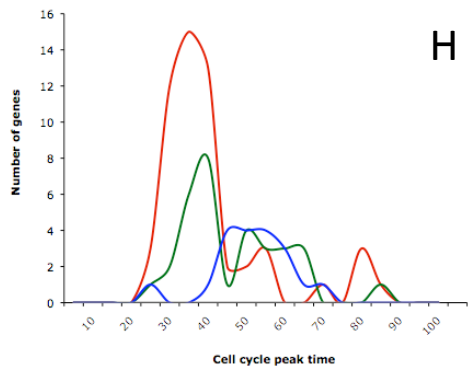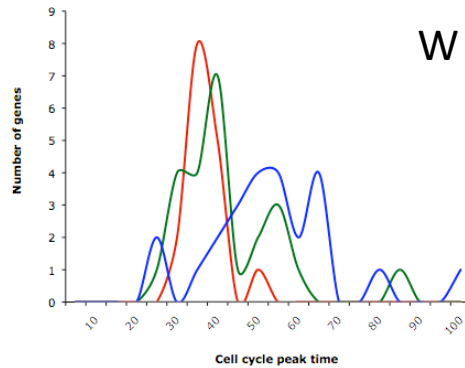

Deactivation

Supplement: Additional file 6 — Cell cycle distributions of predicted targets according to the timing of deactivation of expression from [39]. Letter and color keys as in Additional file 4. [file gb-2010-11-6-r67-S6.PDF]
